# Transplant Trial Watch

**DOI:** 10.3389/ti.2024.13211

**Published:** 2024-05-31

**Authors:** John M. O’Callaghan, Keno Mentor

**Affiliations:** ^1^ University Hospitals Coventry and Warwickshire, Coventry, United Kingdom; ^2^ Centre for Evidence in Transplantation, Nuffield Department of Surgical Sciences, University of Oxford, Oxford, United Kingdom

**Keywords:** randomised controlled trial, liver transplantation, hepatorenal syndrome, solid organ transplantation, hospitalization costs

To keep the transplantation community informed about recently published level 1 evidence in organ transplantation ESOT and the Centre for Evidence in Transplantation have developed the Transplant Trial Watch. The Transplant Trial Watch is a monthly overview of 10 new randomised controlled trials (RCTs) and systematic reviews. This page of Transplant International offers commentaries on methodological issues and clinical implications on two articles of particular interest from the CET Transplant Trial Watch monthly selection. For all high quality evidence in solid organ transplantation, visit the Transplant Library: www.transplantlibrary.com.

**Randomised Controlled Trial 1**

Decreased Need for RRT in Liver Transplant Recipients After Pretransplant Treatment of Hepatorenal Syndrome-Type 1 With Terlipressin.

*by Weinberg, E. M., et al. Liver Transplantation 2023 [record in progress].*

## Aims

This *post hoc* analysis of the CONFIRM trial aimed to examine whether terlipressin was effective in reducing the need for renal replacement therapy (RRT) and improving posttransplant outcomes in liver transplant recipients.

## Interventions

Participants in the CONFIRM trial were randomised to receive either terlipressin plus albumin or placebo.

## Participants

300 liver transplant recipients from the CONFIRM trial.

## Outcomes

The main outcomes of interest were the incidence of hepatorenal syndrome-type 1 (HRS-1) reversal, need for RRT (pretransplant and posttransplant), and overall survival.

## Follow-Up

12 months.

## CET Conclusion

*by Keno Mentor*

Hepatorenal syndrome (HRS) resulting in renal dysfunction results in poorer outcomes following liver transplantation (LT). The efficacy of Terlipressin in reducing HRS in liver failure patients was investigated in the CONFIRM trial, which showed significantly improved rates of HRS, but no difference in mortality at 90 days. This *post hoc* analysis of the CONFIRM trial aimed to determine the difference in renal outcomes (pre and post LT) and 1-year survival in patients who had Terlipressin versus those who did not. The analysis found significant improvements in renal outcomes and 1-year survival in the Terlipression group. However, sub-group analysis showed that patients with more severe liver and renal disease showed poorer outcomes with terlipressin use, indicating a need for careful patient selection. Further trials will be required to better define the patient sub-group that will derive the most benefit from Terlipressin therapy.

## Trial Registration

ClinicalTrials.gov—NCT02770716.

## Funding Source

No funding received.

**Randomised Controlled Trial 2**

Cytomegalovirus Related Hospitalization Costs Among Hematopoietic Stem Cell and Solid Organ Transplant Recipients Treated With Maribavir Versus Investigator-Assigned Therapy: A US-Based Study.

*by Schultz, B. G., et al. Transplant Infectious Disease 2024 [record in progress].*

## Aims

The aim of this study was to use the data from the randomised controlled trial, SOLSTICE, to estimate the cytomegalovirus (CMV) related healthcare resource utilization (HCRU) costs of maribavir (MBV) versus investigator-assigned therapy (IAT), among hematopoietic stem cell transplant (HSCT) and solid organ transplant (SOT) recipients.

## Interventions

Participants in the SOLSTICE trial were randomised to either receive IAT or MBV therapy.

## Participants

352 patients that had either HSCT (40%) or SOT (60%).

## Outcomes

The key outcomes were the cost of hospitalisation with IAT versus MBV therapy, and cost difference (i.e., cost savings) with MBV.

## Follow-Up

N/A.

## CET Conclusion

*by Keno Mentor*

CMV infection which is refractory to standard treatment is a challenging clinical problem, resulting in patient morbidity and increased healthcare costs, mainly due to prolonged and repeat admissions. In the SOLSTICE trail, Maribavir was shown to be more effective than standard treatment protocols for refractory CMV infection in post-transplant patients. This *post hoc* analysis of the SOLISTICE trial used trial data to calculate the reduction in healthcare costs that could be achieved by using Maribavir in this patient population. The analysis demonstrated a third to two-thirds reduction in costs over an 8-week period when using Maribavir. Healthcare cost analyses are complex and subject to many assumptions, which the authors acknowledge introduces significant bias. However, the most striking omission from the analysis is the cost of the Maribavir treatment itself, which is significantly higher than standard therapy. With the additional limitation of a short duration of study, the reliability and applicability of the reported cost savings cannot be readily determined.

## Trial Registration

Not reported.

## Funding Source

Industry funded.

## CLINICAL IMPACT SUMMARY

*by John O’Callaghan*

This paper represents further work from the SOLSTICE study, published in 2022. This RCT investigated the treatment of refractory CMV in organ transplant and stem cell transplant recipients. In the previous paper, Maribavir was shown to be significantly better at clearing CMV than standard treatment, with less nephrotoxicity than foscarnet and less myelosuppression than valganciclovir/ganciclovir.

The current paper focusses on the cost-effectiveness of using Maribavir in this patient group (40% stem cell and 60% solid organ recipients). The potential cost savings are predicated not only on the increased effectiveness of Maribavir, but also on the improved safety profile and reduced complications associated. Clinical data inputs were taken from the SOLSTICE study. Daily costs were derived from the Centers for Medicare and Medicaid Services online price database. Facility-level costs reported by each of the participating facilities in the look-up tool were averaged to yield a representative daily cost.

The authors then used annualised mean length of hospital stay for Maribavir and standard treatment groups using length of stay estimates for ICU and non-ICU beds to calculate a mean Per-Patient-Per-Year (PPPY) hospital-care-related cost. The costs presented in the paper do not take into account any difference in the price of Maribavir compared to standard treatments and so should be viewed in that context. The mean PPPY costs of overall hospitalization was lower in the Maribavir group: $67,205 compared to $145,501. From the results of the previous SOLSTICE paper, and the information in this paper, the use of Maribavir in this population is supported in terms of clinical recovery and safety profile. With regards to the cost effectiveness, it is completely possible that any potential reduction in healthcare associated costs is abrogated by a difference in the treatment cost. A weeks’ course of Maribavir currently costs several thousand US dollars.

The paper was funded by Takeda pharmaceuticals USA Inc. and both first authors are employees of Takeda Pharmaceuticals USA Inc., with stocks in the company.

